# Supplementary material for: Alternatively activated monocyte-derived myeloid cells promote extracellular pathogen persistence within pulmonary fungal granulomas
Source: bioRxiv. 2025 May 27:2025.05.23.655817. Preprint. [Version 1] doi: 10.1101/2025.05.23.655817 (PMC12190343; doi:10.1101/2025.05.23.655817)
Supplement: 1 [file NIHPP2025.05.23.655817V1-supplement-1.pdf]

bioRxiv preprint doi: https://doi.org/10.1101/2025.05.23.655817; this version posted May 27, 2025. The copyright holder for this preprint (which was not certified by peer review) is the author/funder. This article is a US Government work. It is not subject to copyright under 17 USC 105 and is also made available for use under a CC0 license.

Table S1: information for antibodies.

**Antibodies**	**Source**	**Identifier**
AF488 anti-mouse ARG1 (clone A1EXF5)	eBioscience	Cat# 53-3697-80
AF488 anti-mouse IgE (clone RME-1)	BioLegend	Cat# 406909
AF488 anti-GFP (polyclonal)	ThermoFisher	Cat# A-21311
AF647 anti-mouse Ly6G (clone 1A8)	BioLegend	Cat# 127609
AF700 anti-mouse CD31 (clone 30090)	BioLegend	Cat# 102444
AF700 anti-mouse EpCAM (clone G8.8)	BioLegend	Cat# 118240
APC anti-mouse CD45 (clone 30-F11)	BioLegend	Cat# 103112
APC-Cy7 anti-mouse CD11c (clone N418)	BioLegend	Cat# 117324

bioRxiv preprint doi: https://doi.org/10.1101/2025.05.23.655817; this version posted May 27, 2025. The copyright holder for this preprint (which was not certified by peer review) is the author/funder. This article is a US Government work. It is not subject to copyright under 17 USC 105 and is also made available for use under a CC0 license.

APC-Cy7 anti-mouse CD49b (clone DX5)	BioLegend	Cat# 108919
APC-Cy7 anti-mouse TCRβ (clone H67-597)	BioLegend	Cat# 109219
APC-Cy anti-mouse IL-17A (clone TC11-18H10.1)	BioLegend	Cat# 506940
BB700 anti-mouse GATA3 (clone L50823)	BD Bioscience	Cat# 566643
BV421 anti-mouse CD3 (clone 17A2)	BioLegend	Cat# 100227
BV421 anti-mouse IL-5 (clone TRFK5)	BioLegend	Cat# 504311
BV421 anti-mouse Foxp3 (clone MF-14)	BioLegend	Cat# 126419
BV421 anti-mouse PD-L2 (clone TY25)	BioLegend	Cat# 107219
BV510 anti-mouse CD103 (clone 2E7)	BioLegend	Cat# 121423
BV510 anti-mouse CD45.1 (clone A20)	BD Bioscience	Cat# 565278
BV510 anti-mouse CD90.2 (clone 30-H12)	BioLegend	Cat# 105335
BV605 anti-mouse CD4 (clone GK1.5)	BioLegend	Cat# 100451
BV605 anti-mouse CD4 (clone RM4-4)	BioLegend	Cat# 116027
BV605 anti-mouse MertK (clone 2B10C42)	BioLegend	Cat# 151517
BV650 anti-mouse Ki67 (clone 11F6)	BioLegend	Cat# 151215
BV711 anti-mouse CD69 (clone H1.2F3)	BioLegend	Cat# 104537
BV711 anti-mouse Ly6C (clone HK1.4)	BioLegend	Cat# 128037
BV711 anti-mouse IFNγ (clone XMG1.2)	BD Bioscience	Cat# 563736
BV785 anti-mouse KLRG1 (clone 2F1/KLRG1)	BioLegend	Cat# 138429
BV786 anti-mouse CD44 (clone IM7)	BD Bioscience	Cat# 563736
BV786 anti-mouse SiglecF (clone E50-2440)	BD Bioscience	Cat# 740956

bioRxiv preprint doi: https://doi.org/10.1101/2025.05.23.655817; this version posted May 27, 2025. The copyright holder for this preprint (which was not certified by peer review) is the author/funder. This article is a US Government work. It is not subject to copyright under 17 USC 105 and is also made available for use under a CC0 license.

FITC anti-mouse CD8 (clone S18018E)	BioLegend	Cat# 162313
FITC anti-mouse IgG1 (clone RMG1-1)	BioLegend	Cat# 406605
PE anti-human CD2 (clone RPA-2.10)	BioLegend	Cat# 300207
PE anti-human CD4 (clone RPA-T4)	BioLegend	Cat# 300508
PE anti-mouse T-bet (clone 4B10)	BioLegend	Cat# 644809
PE-Cy5 anti-mouse CD64 (clone X54-5/7.1)	BioLegend	Cat# 139332
PE-Cy7 anti-mouse CD11c (clone N418)	BioLegend	Cat# 117317
PE-Cy7 anti-mouse iNOS (clone CXNFT)	eBioscience	Cat# 25-5920-80
PE-Cy7 anti-mouse Ly6G (clone 1A8)	BioLegend	Cat# 127617
PE-Cy7 anti-mouse CD301b (MGL2) (clone URA-1)	BioLegend	Cat# 146807
PE-Cy7 anti-mouse RORγt (clone B2D)	eBioscience	Cat# 25-6981-82
PerCP-Cy5.5 anti-mouse MHCII (clone M5/114.15.2)	BioLegend	Cat# 107625
R718 anti-mouse IL-13 (clone W19-895)	BD Bioscience	Cat# 569945
Anti-Glucuronoxylomannan (GXM) (clone 18B7)	Sigma-Aldrich	Cat# MABF2069
InVivoMab anti-mouse CD4 (clone GK1.5)	Bio X Cell	Cat# BE0003-1
InVivoMab rat IgG2b isotype control (clone LTF-2)	Bio X Cell	Cat# BE0090
